# A rare hepatic mass in an Italian resident

**DOI:** 10.1186/s12876-020-01440-7

**Published:** 2020-09-11

**Authors:** Matteo Borro, Giuseppe Murdaca, Monica Greco, Simone Negrini, Maurizio Setti

**Affiliations:** 1 Department of Internal Medicine (DiMI), Clinical Immunology Unit, University of Genoa and Policlinico San Martino, Viale Benedetto XV, 6 -, 16132 Genova, Italy; 2 Department of Internal Medicine, San Paolo Hospital, Via Genova 30 -, 17100 Savona, Italy

**Keywords:** Amebic liver abscess, Sepsis, Amebiasis, High-risk behavior, Multiple pseudo-nodules, Portal vein thrombosis

## Abstract

**Background:**

Amebiasis is a rare condition in developed countries but epidemiologically growing. Clinical manifestation may range from asymptomatic to invasive disease, amoebic liver abscess being the most common manifestation. We report a peculiar case of left hepatic amoebic liver abscess in a patient without a well-known source of infection and presenting with left portal vein thrombosis.

**Case presentation:**

Patient, working as longshoreman, presented with complaints of remittent-intermittent fever lasting from 2 weeks. Physical examination was normal. Blood tests showed mild anemia, neutrophilic leukocytosis and elevated inflammation markers. Chest x-rays was normal. Abdominal ultrasound showed multiple hypoechoic liver masses. CT-scan of abdomen showed enlarged left liver lobe due to the presence of large abscess cavity along with thrombosis of left portal vein. The indirect hemagglutination test for the detection of antibodies to *Entamoeba histolytica* (Eh) was positive. Ultrasound-guided percutaneous drainage revealed “anchovy sauce” pus. Metronidazole and a follow up imaging at 3 months showed resolution of abscess cavity.

**Conclusion:**

This case shows that amoebic liver abscess is possible even in first world country patients without travel history. Left sided abscess and portal vein thrombosis are rare and hence reported.

## Background

*Entamoeba histolytica* (Eh) is a protozoan parasite with a simple lifecycle that can exists either in the cyst form that is ingested or in the amoeboid trophozoite stage that is responsible for the disease. Infection is usually taken with ingestion of the cysts in food or water previously contaminated by human faeces [1, 2]. Eh is distributed throughout the world and it represents a health risk in those places where barriers between human faeces and water or food are not adequate [1]. Amoebiasis is the third most common cause of death from parasitic diseases, after schistosomiasis and malaria [3]. Eh infection is endemic in several developing countries like India, Africa, Mexico, Central America, South America and Indonesia. Clinical manifestations may range from asymptomatic disease, that is the most common presentation, to invasive intestinal and extra-intestinal diseases. Extra-intestinal manifestations are less frequent and characterized by a negative stool antigen detection. Amoebic liver abscess (ALA) is the most common extra-intestinal site of infection [4], but it occurs in less than 1% of Eh infections [5]. Among symptoms, diarrhea, fever and right-upper quadrant pain and tenderness are the most common ones but also cough, chest pain and weight loss can be seen [1]. In developed countries amoebiasis is a rare condition but epidemiologically growing, especially in high-risk groups such as travelers to and immigrants from endemic areas, men who have sex with men [6] and patients with cell-mediated immunodeficiencies, e.g. HIV infection [7]. Here we report a peculiar clinical case of ALA characterized by the absence of an evident source of infections, a silent clinical presentation and the presence of an extremely rare complication.

## Case presentation

**Fig. 1:**
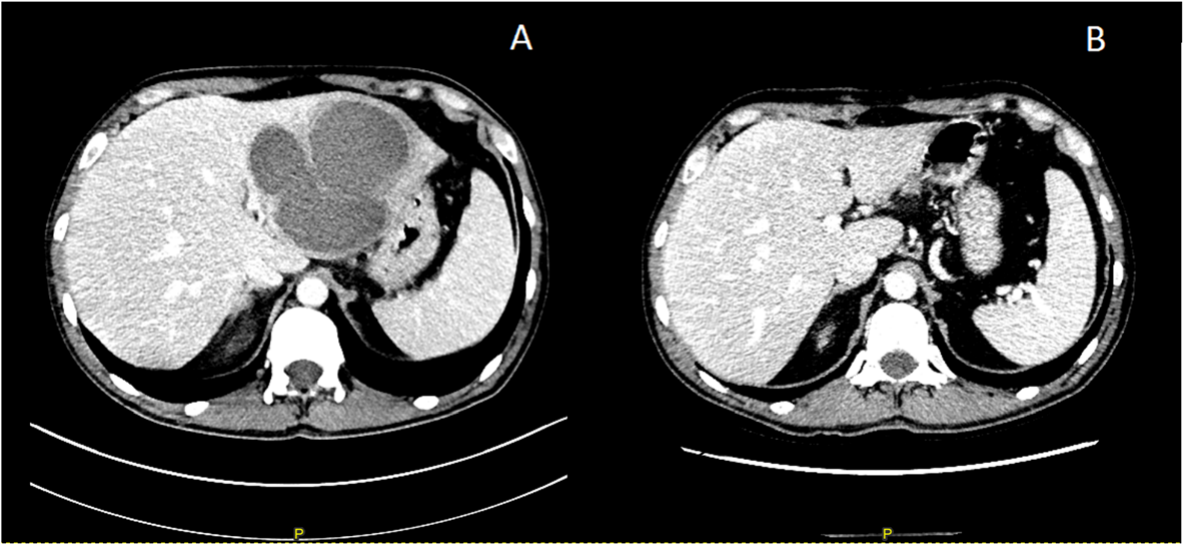
Panel **a**: CT-scan showing enlarged left liver lobe due to the presence of a single large loculated abscess cavity. Panel **b**: CT-scan performed 3 months later showing a normal liver structure

**Fig. 2:**
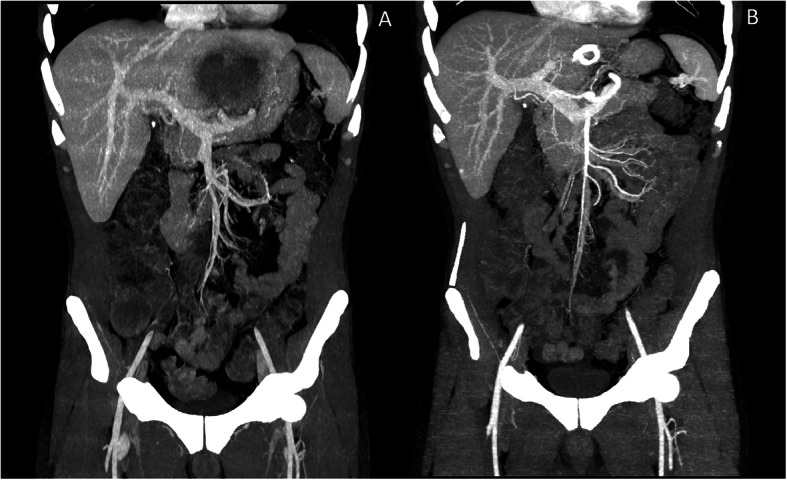
Panel **a**: Coronal image with Maximum Intensity Projection reconstruction showing signs of left portal vein thrombosis. Panel **b**: Coronal image with Maximum Intensity Projection reconstruction performed 3 months later and showing a restored left portal vein flow

A 44-year-old European longshoreman, with previous history of alcohol abuse, came to our clinic for remittent-intermittent fever lasting from 2 weeks. His only complaint was worsening fatigue. His general practitioner started a broad-spectrum antibiotic therapy (Amoxicillin/clavulanic acid 875/125 mg/8 h) without any benefit. Physical examination was completely negative and no obvious sources of infection could be found. Laboratory tests revealed a grade-I mild anemia (Hb 11,7 g/dL), neutrophilic leukocytosis (neutrophils count 16,1 × 10^9^/L) without peripheral eosinophilia, elevated C-reactive protein (337 mg/L), alkaline-phosphatase, γ-glutamyl-transferase, ferritin and procalcitonin (0,86 μg/L) level. Urinalysis was normal and hemocultures, urine culture, serum (1–3)-beta-D-glucan levels, Human Immunodeficiency Virus antibody test, hepatitis and echinococcal serology and antibodies against cytomegalovirus and toxoplasma were all negative. *Mycobacterium tuberculosis* infection was excluded by a negative Quantiferon TB-Test. Chest x-ray was normal. Empiric antimicrobial therapy with Piperacilline/Tazobactam 4 g/0,5 g/8 h was started. Abdominal ultrasound showed multiple hypoechoic liver masses that needed further investigations. Abdominal CT Scan with contrast showed enlarged left liver lobe due to the presence of a single large loculated abscess cavity along with thrombosis of left portal vein. Abscess size was 10 cm × 9,9 cm × 6,6 cm. (Fig. 1, Panel a). Thrombosis was suggested by a shrinking caliber and a global reduced vascularization of the left portal branch (Fig. 2, Panel a). This finding was in close relationship with the gastric wall. Clinical conditions significantly worsened with hypotension, lethargy and uncontrolled fever. Laboratory tests showed grade-II moderate anemia, thrombocytosis and increased transaminase levels. Tumor markers were completely negative. In agreement with antimicrobial stewardship, broad-spectrum intravenous antibiotic therapy with meropenem 2 g/8 h and vancomycin 1 g/12 h was started [8]. The indirect hemagglutination test for the detection of antibodies to Eh was positive, suggesting the diagnosis of ALA, but stool Eh antigen, interestingly, resulted negative. Meanwhile, in order to confirm diagnosis and accordingly to published materials that refer large left-lobe abscesses at high risk of pericardial or peritoneal rupture as well as accelerated and worsening clinical course suggestive for imminent rupture [1, 5], an ultrasound-guided percutaneous drainage revealed “anchovy sauce” pus. In drainage fluid, bacterial cultures were negative thus excluding the presence of pyogenic superinfection, but the finding of a positive Eh antigen confirmed the diagnosis of ALA. I.V. metronidazole 15 mg/kg was started and patient showed a rapid clinic and laboratory improvement that leaded to his discharge from hospital. Antibiotic therapy was then withdrawn in metronidazole 500 mg p.o./8 h for 7 days and followed by paromomycin 30 mg/kg for 7 days. Three-months-later CT-scan and sonography showed resolution of abscess cavity (Fig. 1, Panel b) and restored left portal vein flow (Fig. 2, Panel b).

## Discussion and conclusion

Amebiasis is a rare condition in developed countries, even in high-risk subgroups. The presence of liver abscess is usually suggested by clinical history and signs and/or symptoms of the disease. ALA usually comes from hematic circulation, probably via the portal circulation, after amoebic trophozoites have breached the colic mucosa [1]. Interestingly, our case showed three major peculiarities. First, exceptional indolence until severe hepatic diffusion: indeed, left-side lobe abscess localizations are much less frequent and less symptomatic than right-side ones, that usually present prodromic diarrhea, hypochondrial pain and hepatomegaly [5]. Second, and more important, our patient did not refer travels abroad, nor risky-behavior: he could only have somehow been infected during his job as longshoreman in the industrial port. For sure, globalization and easier way to travel have contributed to the emergence of specific parasitic diseases in novel geographical areas and in workers normally considered not at risk [9]. Third, the presence of thrombosis in the left portal vein. Because of abscess compression and mass effect on adjacent structures, extrinsic obstruction and subsequent reduced blood flow combined with the inflammatory environment due to the ongoing infection can lead to vein thrombosis, in agreement with Virchow’s triad. Thrombosis of inferior and/or intrahepatic vena cava is a very rare complication of ALA only described in single case reports regarding patients born in endemic areas [10–14] or with well-determined source of infection [15]. Thrombosis of the portal vein and/or its ramifications is extremely rare but can be possible too [16, 17]. As suggested in literature, antibiotic therapy coupled with percutaneous drainage is the mainstay of management. Specifically, percutaneous drainage should be performed when abscess are larger than 4 cm [18]. Moreover, percutaneous drainage alone in giant pyogenic liver abscess has been demonstrated to be a safe and sufficient treatment with a very low rate of complications [19]. To the best of our knowledge this is the first report of left portal vein thrombosis consequent to ALA in a patient who lives in a Western Country without any obvious risk factor. In conclusion, in developed countries, clinicians should be aware of ALA as a potential diagnosis in cases of fever of unknow origin and unresponsive to broad-spectrum antibiotic-therapy, especially in patients with history of travels, high-risk behavior and, interestingly, jobs like worker in ports, airports and frontiers, not commonly considered at risk for amoebic infections.

## Data Availability

Not applicable.

## Abbreviations

Eh: *Entamoeba histolytica*
ALA: Amoebic liver abscess
